# Professor Tarnier

**Published:** 1898-02

**Authors:** 


Professor Tarnier, the distinguished French obstetrician, died at Paris, November 23,1897, in the seventieth year of his age. His name will go down to posterity as the inventor of the axis-traction forceps—a distinctive contribution to the obstetric armamentarium.

